# Clinical and genetic characteristics of head and neck paragangliomas from China featuring Sino-European comparisons

**DOI:** 10.1210/jendso/bvag062

**Published:** 2026-04-15

**Authors:** Jun Liu, Yingxian Pang, Jianing Yue, Yanting Shen, Jing Zhang, Minghao Li, Xiaowen Xu, Bin Chen, Jue Yang, Daqiao Guo, Jing Wang, Henri J L M Timmers, Christina Pamporaki, Henricus P M Kunst, Longfei Liu, Zhiqiang Lu, Zhiyin Zhang, Susan Richter, Jingjing Jiang

**Affiliations:** Department of Endocrinology and Metabolism, Zhongshan Hospital Fudan University, Shanghai 200032, China; Department of Urology, Xiangya Hospital, Central South University, Changsha 410008, China; Department of Vascular Surgery, Zhongshan Hospital Fudan University, Shanghai 200032, China; Department of Endocrinology and Metabolism, Zhongshan Hospital Fudan University, Shanghai 200032, China; Department of Endocrinology and Metabolism, Zhongshan Hospital Fudan University, Shanghai 200032, China; Department of Urology, Xiangya Hospital, Central South University, Changsha 410008, China; Department of Urology, Xiangya Hospital, Central South University, Changsha 410008, China; Department of Vascular Surgery, Zhongshan Hospital Fudan University, Shanghai 200032, China; Department of Vascular Surgery, Zhongshan Hospital Fudan University, Shanghai 200032, China; Department of Vascular Surgery, Zhongshan Hospital Fudan University, Shanghai 200032, China; Department of Pathology, Xiangya Hospital, Central South University, Changsha 410008, China; Department of Internal Medicine, Radboud University Medical Centre, 6525 GA Nijmegen, The Netherlands; Department of Medicine III, Medical Faculty Carl Gustav Carus, University Hospital Carl Gustav Carus, Technische Universität Dresden, 01307 Dresden, Germany; Department of Otorhinolaryngology, Dutch Academic Alliance Skull Base Pathology, Radboud University Medical Center, 6525 GA Nijmegen, The Netherlands; Department of Otorhinolaryngology, Dutch Academic Alliance Skull Base Pathology, Maastricht University Medical Center+, 6229 HX Maastricht, The Netherlands; Department of Urology, Xiangya Hospital, Central South University, Changsha 410008, China; Department of Endocrinology and Metabolism, Zhongshan Hospital Fudan University, Shanghai 200032, China; Department of Endocrinology and Metabolism, Zhongshan Hospital Fudan University, Shanghai 200032, China; Auckland Cancer Society Research Centre, School of Medical Sciences, University of Auckland, Auckland 1142, New Zealand; Department of Endocrinology and Metabolism, Zhongshan Hospital Fudan University, Shanghai 200032, China

**Keywords:** head and neck paragangliomas, genetics, Chinese ancestry

## Abstract

**Context:**

Head and neck paragangliomas (HNPGLs) are tumors of parasympathetic origin, predominantly associated with pseudohypoxia and pathogenic variants (PVs) in succinate dehydrogenase (SDH) subunits, mainly based on studies in Europeans/Americans.

**Objective:**

This work aimed to assess the genetics of HNPGLs in a large Chinese cohort and compare them with European cases.

**Methods:**

A retrospective cohort study was conducted of HNPGLs from 2 Chinese tertiary-care centers and 2 European centers. Participants included 222 Chinese and 205 European HNPGL patients. Main outcome measures included clinical presentation and genetic status assessed via next-generation sequencing of tumor/blood samples.

**Results:**

The Chinese HNPGL cohort consisted mainly of carotid body HNPGLs (86.9%), fewer jugulotympanic (12.2%), and no vagal HNPGLs. Jugulotympanic HNPGLs showed higher recurrence than carotid body HNPGLs (30.4% vs 8.5%; *P* = .009). PVs were detected in 62.2% of Chinese patients, predominantly in *SDHx* genes. PV frequency was higher in carotid body than jugulotympanic HNPGLs (65.8% vs 37%; *P* = .004), and in more male than female patients (75.9% vs 54.0%; *P* = .001). Compared to those without *SDHx* PVs, *SDHx* PVs carriers were younger (41.0 vs 49.0; *P* < .001), with more multifocal tumors (20.8% vs 7.6%; *P* = .007) and less often female (53.1% vs 76.1%; *P* < .001). Compared with Europeans, Chinese patients were younger (45.0 vs 54.3; *P* < .001), with fewer multilocal HNPGLs (0.9 vs 13.7%; *P* < .001), lower rates of recurrence (13.7% vs 28.0%; *P* = .007), but similar metastasis rates (3.9% vs 5.0%; *P* = .668).

**Conclusion:**

This is the first report of HNPGLs in a large Chinese cohort. Clinical presentation and practice as well as ancestry all likely contribute to the differences observed between Chinese and European patients.

Head and neck paragangliomas (HNPGLs) are rare nonepithelial neuroendocrine neoplasms predominantly arising from parasympathetic ganglia. These tumors account for only 3% to 5% of all paragangliomas (PGLs), with an estimated overall prevalence of 0.3 to 1 per 100 000 individuals [1]. HNPGLs are most frequently found in the carotid body, the jugulotympanic region (ie, jugular foramen and middle ear), and less often around the vagal nerve. Unlike other PGLs, the majority of HNPGLs are nonfunctional and thus the clinical presentation is mainly determined by local mass effects and varies depending on anatomical site [2].

The genetics of PGLs have been extensively studied, and susceptibility genes are broadly categorized into 3 distinct clusters. Cluster 1 genes, characterized by pseudohypoxia, are categorized into 2 subgroups: cluster 1A comprises genes encoding all succinate dehydrogenase (SDH) subunits and the assembly factor *SDHAF2* (together *SDHx*), *FH*, and other genes associated with the Krebs cycle, while cluster 1B encompasses predominantly *EPAS1* and *VHL*. Cluster 2 genes involve dysregulation of the kinase signaling pathway (*RET*, *NF1*, *HRAS*, *FGFR1*, *TMEM127*, etc), and cluster 3 genes are associated with alterations in the WNT signaling pathway (*CSDE1* and *MAML3* fusions) [3, 4]. The risk of HNPGLs is heavily influenced by genetics, and syndromic presentations of HNPGLs are mainly associated with pathogenic variants (PVs) in *SDHx* [5]. SDH malfunction drives tumorigenesis via activating the HIF2α signaling pathway [6]. High recurrence and metastasis risks have been described in European/North American HNPGLs [7]. It was suggested that PVs in *SDHD* are the most common and correlate with multifocality of HNPGLs, while PVs in *SDHB* are less prevalent but more associated with metastasis [5].

A recent analysis of genetic differences between Asians and Europeans/North Americans with PGLs noted a lack of data associated with HNPGLs in Asian individuals [8]. The present study addresses this shortcoming and investigates clinical and genetic characteristics of Chinese patients with HNPGLs. To date, it remains unclear whether conclusions based on findings in Europeans/North Americans also apply to Chinese patients. By taking advantage of a large cohort of patients with HNPGLs from China, as well as a large cohort from Europe, the present study had 2 objectives: (1) to document clinical and genetic features of Chinese patients with HNPGLs; and (2) to compare Chinese HNPGLs with European HNPGLs.

## Materials and methods

### Study design and participants

**Figure 1:**
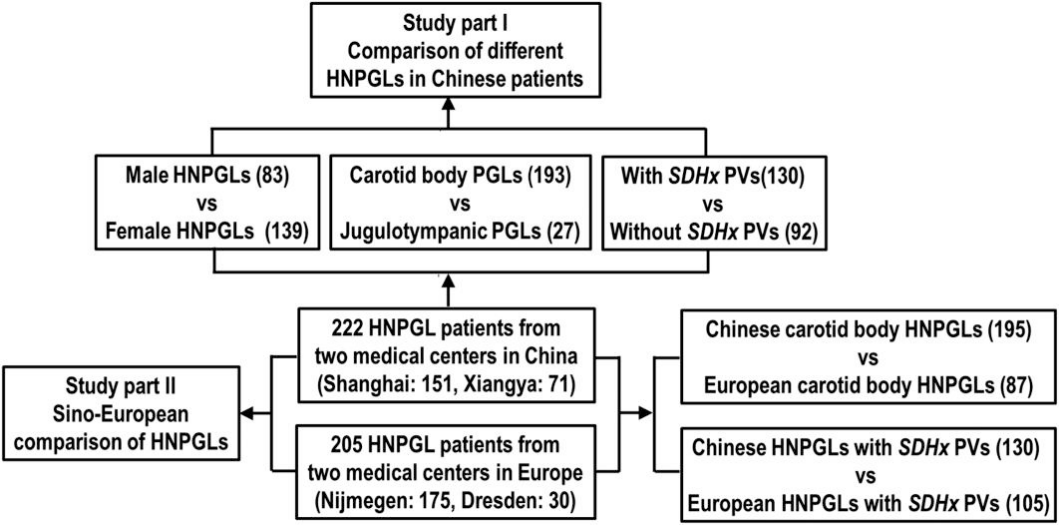
Study flow of HNPGL cohort. HNPGL, head and neck paraganglioma; PV, pathogenic variant.

This retrospective cohort study (Fig. 1) was based on an HNPGL cohort that includes 151 Chinese patients from Zhongshan Hospital, Fudan University, and 71 patients from Xiangya Hospital, Central South University. These patients were diagnosed and treated between January 1, 2015 and December 31, 2021. All Chinese HNPGLs were pathologically confirmed, with frozen or paraffin-embedded samples available for genetic testing. The study also included a cohort of 205 European patients with HNPGLs from the Netherlands and Germany that was part of a previous publication [7]. The study conforms to the Declaration of Helsinki and Good Clinical Practice guidelines. All patients were recruited under study protocols approved by the ethics committees of each participating hospital. Written informed consent for genetic analyses and use of existing clinical data was obtained from all patients.

### Data collection

For all Chinese patients, we collected patient data including age at initial diagnosis, sex, previous and family history of HNPGLs, presence of syndromic presentation, location of tumors, size of primary tumors measured by pathology, clinical presentation of HNPGLs, metastasis, locoregional recurrence, and follow-up information. For Chinese patients, presurgical biochemical testing for catecholamine excess was not systematically performed. Syndromic presentation was defined as bilateral or multiple tumors, or those typical of von Hippel-Lindau (VHL) syndrome, neurofibromatosis type 1, multiple endocrine neoplasia type 2, Pacak-Zhuang syndrome, etc. Multilocal HNPGL was defined as HNPGLs in more than one anatomic head and neck location, excluding bilateral tumors within the same region. Multifocal HNPGL is composed of both bilateral HNPGL and multilocal HNPGL. Chinese patients were followed at intervals of 1 to 2 years by computed tomography (CT) angiography, and those with impaired renal function were followed by ultrasound or noncontrast CT instead. The images were centrally reviewed and cross-checked by the surgical team. In the European cohort, for patients with PVs in *SDHx*, annual clinical and biochemical follow-up was performed along with sonography of the head and neck and/or magnetic resonance imaging (MRI) of the neck. In addition, whole-body MRI or 68Ga-DOTATATE imaging was performed every 3 to 5 years. For patients without PVs in *SDHx*, annual follow-up with sonography or MRI of the head and neck was performed for the first 3 years, followed thereafter by longer intervals on an individualized basis. Recurrence was defined as the presence of new locoregional tumors after at least 1 year of radical surgical removal of the initial tumor. Metastasis was defined as the presence of PGLs in nonchromaffin tissues identified by imaging or pathology. We also recorded presence of PVs in known susceptibility genes for PGL and the type of PV (somatic or germline) based on genetic screening.

### Genetic testing

Genetic testing was performed using formalin-fixed, paraffin-embedded tumor specimens or fresh frozen tumor tissue, using a customized next-generation sequencing (NGS) panel covering *SDHA*, *SDHB*, *SDHC*, *SDHD*, *SDHAF2*, *IDH1*, *IDH2*, *FH*, *MDH2*, *VHL*, *EPAS1*, *EGLN1*, *EGLN2*, *RET*, *NF1*, *HRAS*, *FGFR1*, *MAX*, *TMEM127*, *DNMT3A*, *SLC25A11*, and *H3F3A*. All PVs identified by NGS were confirmed by Sanger sequencing and tested in peripheral blood if available. For all patients from Europe, germline testing was performed by NGS panels and confirmed by Sanger sequencing [7]. PVs refer to variants classified as pathogenic or likely pathogenic according to American College of Medical Genetics and Genomics/Association for Medical Pathology guidelines [9].

### Immunohistochemistry

Immunohistochemistry for tyrosine hydroxylase (TH) and phenylethanolamine *N*-methyltransferase (PNMT) was performed on 4-µm sections from formalin-fixed, paraffin-embedded tumors. Slides were incubated overnight with rabbit anti-TH polyclonal antibody (Proteintech catalog No. 25859-1-AP, RRID: AB_2716568, 1/300 dilution) or rabbit anti-PNMT polyclonal antibody (Proteintech catalog No. 13217-1-AP, RRID: AB_2167781, 1/300 dilution), followed by incubation with secondary antibody (horseradish peroxidase–conjugated goat anti-rabbit immunoglobulin G polyclonal antibody, Huabio catalog No. HA1001, RRID:AB_2819166, 1/200 dilution) for 30 minutes.

### Statistical analysis

Continuous variables with normal distribution were analyzed using the *t* test for 2-group comparisons. For nonnormally distributed continuous variables, the Mann-Whitney *U* test was employed for intergroup comparisons, with 95% CIs calculated using the Wilson score method. Categorical variables were assessed using either the χ^2^ test or Fisher exact test for frequency comparisons. A 2-tailed *P* value less than .05 was considered statistically significant for all analyses. All statistical analyses were performed using RStudio (version 2024.12.1 + 563, Posit Software, PBC).

## Results

### Clinical characteristics of the Chinese head and neck paragangliomas cohort

Among the Chinese cohort, the median age of first diagnosis was 45.0 years, and nearly two-thirds of the patients (62.6%) were female. Most patients presented with carotid body HNPGLs (86.9%) and a minority with jugulotympanic HNPGLs (12.2%), while none of the patients had vagal HNPGLs. Bilateral HNPGLs were present in 14.4% of patients with no difference between the sexes (Tables 1 and 2). Only 2 patients had multilocal HNPGLs, that is, HNPGLs in more than one location. One of them had a left carotid body HNPGL, bilateral jugular HNPGLs, and an HNPGL at the right cerebellopontine angle. The other patient had concurrent carotid body HNPGL and thyroid PGL. Five patients presented with solitary/bilateral HNPGLs and additional non-HNPGLs. Syndromic presentation was observed in 15.8% of patients, predominantly related to bilateral HNPGLs (see Table 1). Recurrence occurred in 13.7% of patients, while metastasis was rare and observed in 3.9% of patients during a median follow-up period of 84 months (see Table 1).

**Table 1:** Clinical characteristics of the Chinese and European head and neck paraganglioma cohort

	Chinese		European		
Feature	N or median	Percentage or 95% CI	N or median	Percentage or 95% CI	*P*
**Total**	222		205		
**Sex, female**	139	62.6%	136	66.3%	.421
**Age, y**	45.0	42.0-48.0	54.3	51.2-57.2	<.001
**Tumor location**					
Carotid body	193	86.9%	64	31.2%	<.001
Jugulotympanic	27	12.2%	90	43.9%	<.001
Vagal	0	0%	14	6.8%	<.001
Multifocality	34	15.3%	67	32.7%	<.001
Bilateral	32	14.4%	32	15.6%	.730
Multilocal HNPGLs*^a^*	2	0.9%	28	13.7%	<.001
Additional PGLs	5	2.3%	8	3.9%	.321
**Tumor characteristics**					
Tumor volume, cm^3^*^b^*	7.9	6.50-9.40	6.6	3.5-9.7	.015
Syndromic presentation	35	15.8%	46	22.4%	.079
Recurrence	18/131	13.7%	28/100	28.0%	.007
Metastasis	5/127	3.9%	9/181	5.0%	.668
Follow-up time, mo*^c^*	84	42.0-90.0	95	78.0-111.0	.043
**PV-positive tumors**					
Cluster 1A PVs*^d^*	132	59.5%	106	51.7%	.107
*FH*	2	0.9%	0	0.0%	.500
*SDHx*	130	58.6%	106	51.7%	.155
*SDHA*	7	3.2%	9	4.4%	.501
*SDHAF2*	6	2.7%	13	6.3%	.068
*SDHB*	33	14.9%	12	5.9%	.002
*SDHC*	10	4.5%	6	2.9%	.391
*SDHD*	74	33.3%	65	31.7%	.720
Somatic PVs	6	2.7%	—	—	
*EPAS1*	2	0.9%	—	—	
*VHL*	3	1.4%	—	—	
*HRAS*	1	0.5%	—	—	

Abbreviations: HNPGL, head and neck paraganglioma; PGLs, paragangliomas; PV, pathogenic variant (including germline and somatic for Chinese and germline for European).

^*a*^Multilocal is defined as more than one HNPGL location (excluding bilateral of the same region).

^*b*^For those with non-HNPGLs, only HNPGL was calculated.

^*c*^Only patients who had at least 12 months of postoperative follow-up were included in the analysis.

^*d*^In the Chinese cohort, 72 of the 132 cases were confirmed germline PVs, while blood DNA was unavailable for 50 cases. No somatic PVs in *SDHx* or *FH* were confirmed. In the European cohort, all PVs were confirmed to be germline.

**Table 2:** Clinical characteristics of Chinese head and neck paragangliomas in male vs female patients

	Male		Female		
	N or median	Percentage or 95% CI	N or median	Percentage or 95% CI	*P*
**Total**	83		139		
**Age, y**	44	41.0-50.0	46	41.0-49.0	.823
**Tumor characteristics**					
Carotid body	76	91.6%	117	84.2%	.114
Jugulotympanic	7	8.4%	20	14.4%	.189
Multifocality	16	19.3%	18	12.9%	.205
Bilateral	16	19.3%	16	11.5%	.111
Multilocal HNPGLs	0	0.0%	2	1.4%	.530
Additional PGLs	2	2.4%	3	2.2%	≥.999
Tumor volume, cm^3^*^a^*	8.9	6.10-11.5	7.9	5.6-9.4	.353
Syndromic presentation	18	21.7%	17	12.2%	.061
Family history	6	7.2%	4	2.9%	.181
Apparently sporadic	61	73.5%	120	86.3%	.017
Recurrence	5/52	9.6%	13/79	16.5%	.266
Metastasis	0/49	0%	5/78	6.4%	.156
Follow-up time, mo*^b^*	84	70.8-91.2	76.8	61.2-91.2	.568
**PV-positive tumors**	63	75.9%	75	54.0%	.001
*SDHx*	61	73.5%	69	49.6%	<.001
*SDHB*	20	24.1%	13	9.4%	.003
*SDHD*	32	38.6%	42	30.2%	.202
*Non-SDHx*	2	2.4%	6	4.3%	.713
**Negative** *^c^*	17	20.5%	51	36.7%	.011

Abbreviations: HNPGL, head and neck paraganglioma; PV, pathogenic variant.

^*a*^For those with non-HNPGLs, only HNPGL was calculated.

^*b*^Only patients who had at least 12 months of postoperative follow-up were included in the analysis.

^*c*^Variants of unknown significance were not included.

The median age of diagnosis was similar between male and female patients (age 44 vs 46; *P*  *=* .823) (see Table 2). The only 2 multilocal HNPGLs were both found in women. Syndromic presentation tended to be more common in male than female patients (21.7% vs 12.2; *P* = .061). Consistently, the proportion of apparently sporadic tumors (ie, solitary tumor, without syndromic presentation or family history) was lower in male than in female patients (73.5% vs 86.3%; *P* = .017) (see Table 2). Locoregional recurrence was present in 9.6% of male and 16.5% of female patients, respectively (*P* = .266), and all 5 patients with metastatic disease were female (0% vs 6.4%; *P* = .156) (see Table 2).

Female patients accounted for 60.6% of carotid body HNPGLs and 74.1% of jugulotympanic HNPGLs (*P* = .177) (Table 3). Bilateral tumors accounted for 15.0% of carotid body HNPGLs and 7.4% of jugulotympanic HNPGLs (*P* = .385) (see Table 3). Locoregional recurrence rates were nearly 4 times higher in jugulotympanic than carotid body HNPGLs (30.4% vs 8.5%; *P* = .009), though metastasis was rare and comparable between carotid body and jugulotympanic HNPGLs (3.8% vs 4.8%; *P* = 1.000) (see Table 3).

**Table 3:** Clinical and genetic characteristics of carotid body head and neck paragangliomas (HNPGLs) vs jugulotympanic HNPGLs in Chinese patients

	Carotid body HNPGL*^a^*		Jugulotympanic HNPGL		
	N or median	Percentage or 95% CI	N or median	Percentage or 95% CI	*P*
**Total**	193		27		
**Sex, female**	117	60.6%	20	74.1%	.177
**Age, y**	44	41.0-48.0	48	43.0-53.0	.141
**Tumor volume, cm^3^**	8.8	6.5-10.5	5.3	2.7-10.5	.134
**Bilateral**	29	15.0%	2	7.4%	.385
**Additional PGLs**	4	2.1%	1	3.7%	.484
**Syndromic presentation**	31	16.1%	2	7.4%	.387
**Family history**	9	4.7%	1	3.7%	≥.999
**Apparently sporadic**	157	81.3%	24	88.9%	.429
**Recurrence**	9/106	8.5%	7/23	30.4%	.009
**Metastasis**	4/104	3.8%	1/21	4.8%	≥.999
**Follow-up time, mo** *^b^*	80.4	68.4-88.8	86.4	52.8-110.4	.272
**PV-positive tumors**	127	65.8%	10	37.0%	.004
*SDHx*	119	61.7%	10	37.0%	.015
*SDHB*	29	15.0%	4	14.8%	≥.999
*SDHD*	69	35.8%	4	14.8%	.030
*Non-SDHx*	8	4.1%	0	0%	.600
**Negative** *^c^*	50	25.9%	17	63.0%	<.001

Abbreviations: PGL, paraganglioma; PV, pathogenic variant.

^*a*^Two patients with multilocal HNPGLs were excluded from analysis.

^*b*^Only patients who had at least 12 months of postoperative follow-up were included in the analysis.

^*c*^Variants of unknown significance were not included.

### Genetic phenotype of Chinese patients with head and neck paragangliomas

In the Chinese cohort, PVs were detected in nearly two-thirds of all patients (62.2%), and most PVs occurred in *SDHx* genes, especially in *SDHD* (33.3%) and *SDHB* (14.9%) (see Table 1). Frameshifts were the most common events in the *SDHD* gene, while missense variants were most frequent in *SDHB* (Supplementary Table S1) [10]. PVs in *SDHA*, *SDHC*, and *SDHAF2* were rare, accounting for 10.4% altogether. The proportion of PV-positive HNPGLs was higher in male than in female patients, due to more *SDHx* PVs present in male patients (see Table 2). Despite comparable proportions of *SDHD* PVs (38.6% vs 30.2%; *P* = .202), the proportion of *SDHB* PVs was higher in male than in female patients (24.1% vs 9.4%; *P* = .003) (see Table 2).

PVs were more frequently identified in patients with carotid body HNPGLs than with jugulotympanic HNPGLs (65.8% vs 37.0%; *P* = .004) (see Table 3). Specifically, PVs in *SDHx* genes were more prevalent in patients with carotid body compared to jugulotympanic HNPGLs, and this was mainly due to a higher proportion of *SDHD* PVs (35.8% vs 14.8%; *P* = .030) (see Table 3). In contrast, the proportions of *SDHB* PVs were comparable between patients with carotid body and jugulotympanic HNPGLs (15.0% vs 14.8; *P* = 1.000) (see Table 3). PVs in *SDHAF2* were present only in patients with carotid body HNPGLs.

In addition to PVs in *SDHx*, rare germline PVs in *FH* and somatic PVs in *EPAS1*, *VHL*, and *HRAS* were detected, all in patients with carotid body HNPGLs (see Table 3). Two patients had germline *FH* PVs, without leiomyoma or renal cell carcinomas. Somatic *EPAS1* PVs were found in 2 patients with apparently sporadic HNPGLs, without any sign of Pacak-Zhuang syndrome. One tumor with an *EPAS1* PV stained weakly positive for TH, while the other was completely negative (Supplementary Fig. S1) [10]. Three patients had HNPGLs with somatic PVs in *VHL* but did not show syndromic presentation or family history suggestive of VHL syndrome. The only PV in a cluster 2 gene was identified in *HRAS*. The tumor was negative for TH and PNMT on immunohistochemistry, implying nonfunctional status (see Supplementary Fig. S1) [10]. In summary, PVs in cluster 1A genes, mainly in *SDHx*, were the most frequent in Chinese patients with HNPGLs, with PVs in cluster 1B and cluster 2 genes being present in small proportions.

### Characteristics of Chinese patients with head and neck paragangliomas with and without pathogenic variants in *SDHx*

*SDHx* PVs were found in 58.6% of patients (see Table 1). The proportion of females was significantly higher in HNPGLs without *SDHx* PVs compared to those with *SDHx* PVs (76.1% vs 53.1%; *P* < .001) (Table 4). Among those without *SDHx* PVs, apparently sporadic HNPGLs were more common in female than male patients (94.3% vs 68.2%; *P* = .003). The median age of diagnosis among patients with *SDHx* PVs was 8 years younger than that of patients without *SDHx* PVs (*P* < .001). More carotid body HNPGLs (91.5% vs 80.4%; *P* = .016) but fewer jugulotympanic HNPGLs (7.7% vs 18.5%; *P* = .015) were associated with *SDHx* PVs (see Table 4).

**Table 4:** Clinical characteristics of head and neck paragangliomas with vs without pathogenic variant in the *SDHx* genes in Chinese patients

	With *SDHx* PVs		Without *SDHx* PVs		
	N or median	Percentage or 95% CI	N or median	Percentage or 95% CI	*P*
**Total**	130		92		
**Sex, female**	69	53.1%	70	76.1%	<.001
**Age, y**	41.0	38.9-43.3	49.0	46.6-51.4	<.001
**Tumor location**					
Carotid body	119	91.5%	74	80.4%	.016
Jugulotympanic	10	7.7%	17	18.5%	.015
Multifocality	27	20.8%	7	7.6%	.007
Bilateral	26	20.0%	6	6.5%	.005
Multilocal HNPGLs	1	0.8%	1	1.1%	≥.999
Additional PGLs	5	3.8%	0	0.0%	.078
**Tumor characteristics**					
Tumor volume, cm^3^*^a^*	7.9	6.10-9.40	8.9	5.2-11.5	.803
Syndromic presentation	28	21.5%	7	7.6%	.005
Family history	6	4.6%	4	4.3%	≥.999
Apparently sporadic	100	76.9%	81	88.0%	.035
Recurrence	10/82	12.2%	8/49	16.3%	.506
Metastasis	5/79	6.3%	0/48	0.0%	.156
Follow-up time, mo*^b^*	84	70.8-88.8	76.8	60-88.8	.250

Abbreviations: HNPGL, head and neck paraganglioma; PGLs, paragangliomas; PV, pathogenic variant.

^*a*^For those with non-HNPGLs, only HNPGL was calculated.

^*b*^Only patients who had at least 12 months of postoperative follow-up were included in the analysis.

Bilateral HNPGLs and syndromic presentation were almost 3 times more common in patients with than without *SDHx* PVs (20.0% vs 6.5%; *P* = .005 and 21.5% vs 7.6%; *P* = .005, respectively) (see Table 4). Multifocality (bilateral and multilocal) predominantly occurred in patients with *SDHD* PV. Five patients had additional non-HNPGLs, including sympathetic adrenal/extra-adrenal PGLs, and all had PVs in the *SDHD* gene. The proportion of patients with family history was similar between those with and without *SDHx* PVs; this included 4 patients with family history but without PVs identified. Apparently sporadic HNPGLs were less common among those with *SDHx* PVs, compared to those without *SDHx* PVs (76.9% vs 88.0%; *P* = .035). Recurrence rates were comparable between patients with and without *SDHx* PVs. In contrast, all 5 patients with metastasis had PVs in *SDHx*, namely 3 with germline PVs in *SDHD* (1 start-loss, 1 stop-gain, and 1 frameshift), and 2 with germline PVs in *SDHB* (missense variants) (see Table 4).

### Sino-European differences in clinical and genetic characteristics of head and neck paragangliomas

Since we have previously described the effect of ancestry on the characteristics/genetics of PPGLs [8-10], we explored the difference between Chinese and European patients with HNPGLs by comparison with a previously published European cohort [7]. The median age of Chinese patients at diagnosis was nearly 10 years younger than European patients (45.0 vs 54.3; *P* < .001) (see Table 1). Compared to the Chinese cohort, the European cohort was more diversified with more jugulotympanic and vagal HNPGLs (see Table 1). Multifocality was higher in European patients, predominantly due to multilocal HNPGLs being more than 10 times more common in European than in Chinese patients (13.7% vs 0.9%; *P* < .001). The median tumor size in Chinese patients was slightly larger than in European individuals (7.9 cm^3^ vs 6.6 cm^3^; *P* = .015). During a median follow-up period of 84 months in the Chinese and 95 months in the European cohort, respectively (*P* = .043), locoregional recurrence was twice as common in European than in Chinese patients (28.0% vs 13.7%; *P* = .007), though metastasis was comparable (5.0% vs 3.9%; *P* = .668) (see Table 1). The proportion of *SDHx* PV-positive HNPGLs was similar in Chinese and European patients (58.6% vs 51.7%; *P* = .155). Although PVs in the *SDHD* gene were comparable between the two cohorts (33.3% vs 31.7%; *P* = .720), PVs in *SDHB* were more frequent in Chinese than European patients (14.9% vs 5.9%; *P* = .002) (see Table 1). Somatic PVs in cluster 1B and cluster 2 were found only in Chinese patients, as somatic screening was not routinely performed in Europeans.

### Comparison of carotid body head and neck paragangliomas between Chinese and Europeans

Since most of the patients in the Chinese cohort consisted of carotid body HNPGLs, we next compared carotid body HNPGLs between Chinese and Europeans. Chinese patients with carotid body HNPGL were diagnosed younger than Europeans (age 44.0 vs 49.0 years median; *P* = .018). The proportion of bilateral tumors in Chinese patients was only half of that of European patients (15.4% vs 34.5%; *P* < .001). Multilocal HNPGLs were rare among Chinese patients compared to European patients (1% vs 23%; *P* < .001). The median tumor size was larger in European than Chinese patients (12.7 vs 8.8 cm^3^; *P* = .018), caused by a higher percentage of multilocal HNPGLs in the former cohort. Recurrence was nearly 3 times more common in European than in Chinese patients (24.4% vs 8.5%; *P* < .001), though metastasis was not statistically significantly different (7.6% vs 3.8%; *P* = .329) (Table 5). Notably, follow-up times between the cohorts differed significantly.

**Table 5:** Clinical and genetic characteristics of carotid body head and neck paragangliomas in Chinese vs European individuals

	Chinese cohort		European cohort		
	N or	Percentage	N or	Percentage	*P*
	median	or 95% CI	median	or 95% CI	
Total	195		87		
Female sex	119	61.0%	43	49.4%	.085
Age, y	44.0	41.0-47.0	49.0	43.0-53.5	.018
Tumor volume, cm^3^*^a^*	8.8	6.9-10.5	12.7	10.6-18.0	.018
Mutifocality	32	16.4%	50	57.5%	<.001
Bilateral	30	15.4%	30	34.5%	<.001
Multi-local HNPGLs	2	1.0%	20	23.0%	<.001
Additional PGLs	4	2.1%	7	8.0%	.039
Recurrence	11/108	8.5%	10/41	24.4%	<.001
Metastasis	4/106	3.8%	6/79	7.6%	.329
Follow-up time, mo*^b^*	81.6	69.6-88.8	108	84-130.8	<.001
Cluster 1A PV-positive	122	62.6%	65/83	78.3%	.010
*FH*	2	1.0%	0	0	≥.999
*SDHx*	120	61.5%	65/83	78.3%	.007
*SDHA*	6	3.1%	4/83	4.8%	.492
*SDHAF2*	6	3.1%	11/83	13.3%	.001
*SDHB*	29	14.9%	2/83	2.4%	.003
*SDHC*	9	4.6%	2/83	2.4%	.515
*SDHD*	70	35.9%	46/83	55.4%	.003
Negative*^c^*	51	26.2%	18/83	21.7%	.430

Abbreviations: HNPGL, head and neck paraganglioma; PGLs, paragangliomas; PV, pathogenic variant.

^*a*^For those with non-HNPGLs, only HNPGL was calculated.

^*b*^Only patients who had at least 12 months of postoperative follow-up were included in the analysis.

^*c*^Variants of unknown significance were not included.

In contrast to the findings from the complete cohorts, the proportion of *SDHx* PVs were lower in Chinese than in European patients (61.5% vs 78.3%; *P* = .007). PVs in *SDHD* were most common in both cohorts. Nevertheless, the proportion of *SDHD* PVs in Chinese patients was less than that of European patients (35.9% vs 55.4%, *P* = .003) (see Table 5). Contrary to our previous findings in non-HNPGLs [11], the proportion of *SDHB* PVs was higher in Chinese patients than in European patients (14.9% vs 2.4%; *P* = .003). There were fewer *SDHAF2* PVs in Chinese than in European patients (3.1% vs 13.3%; *P* = .001) (see Table 5).

### Sino-European differences in the characteristics of head and neck paragangliomas with pathogenic variants in *SDHx*

Since PVs in *SDHx* were most prevalent in HNPGLs, we further explored the difference between Chinese and European patients with PVs in *SDHx*. No sex bias was found in either Chinese or European patients with PVs in *SDHx*, though Chinese patients were younger than Europeans (41.0 vs 47.2 years median; *P* < .001). Tumor locations differed significantly between Chinese and Europeans because of dominance of carotid body HNPGLs in the Chinese cohort. The rate of bilateral HNPGLs was similar, but compared to Europeans, multilocal HNPGLs were extremely rare in Chinese patents with *SDHx* PVs (29.5% vs 0.8%; *P* < .001). Contrary to the findings in the complete Sino-European cohort (see Table 1), the median size of Chinese HNPGLs from patients with *SDHx* PV was smaller than that of European HNPGLs (7.9 cm^3^ vs 11.3 cm^3^; *P* = .009) (Table 6). Comparisons of recurrence and metastasis rates between Sino-European patients with PVs in *SDHx* reflected similar findings as described for the complete cohorts (see Table 6).

**Table 6:** Clinical characteristics of head and neck paragangliomas with *SDHx* in Chinese vs European cohorts

	Chinese		European		
	N or median	Percentage or 95% CI	N or median	Percentage or 95% CI	*P*
Total	130		105		
Sex, female	69	53.1%	53	50.5%	.692
Age, y	41.0	38.9-43.3	47.2	43-51.2	<.001
Tumor characteristics					
Carotid body	119	91.5%	42	40.0%	<.001
Jugulotympanic	10	7.7%	21	20.0%	.006
Vagal	0	0.0%	10	9.5%	<.001
Multifocality	27	20.8%	61	58.1%	<.001
Bilateral	26	20.0%	30	28.6%	.125
Multi-local HNPGLs	1	0.8%	31	29.5%	<.001
Additional PGLs	5	3.8%	2	1.9%	.465
Tumor volume, cm^3^*^a^*	7.9	6.10-9.40	11.3	7.8-16.6	.009
*SDHx* tumors					
*SDHA*	7	5.4%	9	8.6%	.335
*SDHAF2*	6	4.6%	13	12.4%	.030
*SDHB*	33	25.4%	12	11.4%	.007
*SDHC*	10	7.7%	6	5.7%	.550
*SDHD*	74	56.9%	65	61.9%	.440
Recurrence	10/82	12.2%	16/53	30.2%	.010
Metastasis	5/79	6.3%	9/97	9.3%	.472
Follow-up time, mo*^b^*	84	70.8-88.8	120	96-143	<.001

Abbreviations: HNPGL, head and neck paraganglioma; PGL, paraganglioma; PV, pathogenic variant.

^*a*^For those with non-HNPGLs, only HNPGL was calculated.

^*b*^Only patients who had at least 12 months of postoperative follow-up were included in the analysis.

## Discussion

The present study fills a gap in the understanding of Chinese patients with HNPGLs, as previously only small numbers of Asian patients with HNPGL were reported [8]. Consistent with previous findings in Europeans/North Americans [11-15], a high proportion of women was present among Chinese patients with jugulotympanic HNPGLs. The dominance of PVs in *SDHx* among patients with HNPGLs was confirmed in Chinese patients, with particularly high rates in carotid body HNPGLs. Interestingly, sex differences were observed only in HNPGLs without *SDHx* PVs, implying a sex-specific effect on tumorigenesis of HNPGLs, as previously suggested [8]. The molecular basis for this observation remains to be investigated in future studies. Sex-specific development of HNPGLs could be rooted in a multitude of different factors [16], including hormonal, epigenetic, or metabolic differences or differences in the response to inflammation. Most likely a female-biased signaling cascade that favors cell proliferation may be active in glomus cells. Such a mechanism was described in a *Drosophila* tumor model [17] of the salivary gland.

Although genetic predisposition to HNPGLs is mainly caused by *SDHx* PVs, rare germline PVs in *VHL*, *RET*, *TMEM127*, *FH*, *SLC25A11*, *DNMT3A*, as well as rare somatic PVs in *IDH1* and *IDH2* have also been described [18-24]. Our study adds to this list by identifying rare somatic variants in *HRAS*, *EPAS1*, and *VHL* in Chinese patients with HNPGLs. In the past, the majority of studies about HNPGLs have predominantly relied on germline testing data. This includes the European dataset we have used for comparing features between Chinese and European HNPGLs [7]. Updated consensus guidelines recommend somatic testing as the assay of choice also for HNPGLs [25]. A recent study described two expression clusters in HNPGLs without germline PVs, an *SDHx*-like and a *DNMT3A*-like cluster [26]. The *SDHx*-like cluster consisted of tumors with previously undetected alterations in *SDHx* genes, while the other cluster was characterized by polycomb repressive complex 2 dysfunctions and contained some tumors with *STAG2* mutations. In the Chinese cohort, no PV in *DNMT3A* was identified, but our NGS panel did not include *STAG2*.

In our previous Sino-European study, we described a profound ancestral effect on the clinical and genetic characteristics of non-HNPGLs [11]. In the present study, in the full Sino-European cohort, Chinese patients had more *SDHB* PVs and a younger age of diagnosis, but fewer multilocal HNPGLs and recurrence than European patients. Nevertheless, when confined to carotid body HNPGLs, there were fewer PVs in *SDHx* (predominantly fewer *SDHD* PVs), fewer multifocal HNPGLs, and less recurrence in Chinese compared to European patients with HNPGLs. Contrary to our previous findings in non-HNPGLs, the proportion of PVs in *SDHB* was higher in Chinese than in European patients. This result may reflect a certain bias in the Dutch population of the European cohort, as founder effects have been previously reported [27]. The differences described imply the importance of taking ancestry into consideration for management guidelines. In addition, metastasis is low and only among those with PVs in *SDHx*, so watchful waiting in sporadic HNPGLs could be an option in Chinese patients with asymptomatic tumors without PVs in *SDHx*. Analogous to European data, additional non-HNPGL were observed only in Chinese patients with *SDHx* PVs, making whole-body imaging particularly warranted for these patients, while imaging of the head and neck region is sufficient for those in whom *SDHx* PVs or syndromic presentation are excluded.

In China, the clinical approach toward HNPGLs is more aggressive. Once an HNPGL is identified by imaging, surgical resection is generally recommended. This is mainly based on the fact that HNPGLs are progressive tumors with metastatic potential, and tumor growth is associated with increasing operative complexity, higher intraoperative blood loss, and a greater risk of cranial nerve injury. In general for carotid body HNPGLs, Shamblin type I and II tumors are actively resected. For Shamblin type III tumors, referral to a high-volume tertiary center is common. Indeed, a substantial proportion of the cases included in our cohort were referred from other hospitals specifically for surgical management. In addition, Chinese patients are traditionally more proactive toward tumors with malignant potential and many are reluctant to accept long-term observation. This cultural factor also partly explains the more aggressive approach in China.

In the Chinese cohort, perioperative complications mainly included cranial nerve injury, considerable intraoperative bleeding (>500 mL), and perioperative ischemic stroke. Cranial nerve injury was the most common complication, with an incidence of approximately 20%, most frequently presenting as postoperative dysphagia or voice changes. Notably, many of these symptoms gradually improved during follow-up. Major intraoperative bleeding occurred in less than 5% of cases, and postoperative hematoma in less than 1%. Except for one patient who developed acute airway obstruction leading to asphyxia, all hematomas were successfully managed with prompt reexploration and evacuation. The incidence of perioperative stroke in the Chinese cohort was below 2%. The relatively low rates of severe surgical morbidity might also contribute to the more proactive surgical stance in experienced Chinese centers compared with many European centers. Nevertheless, given the similar metastatic rates and the low risk observed in sporadic, *SDHx*-negative tumors, watchful waiting may be appropriate for asymptomatic Chinese patients, and a more aggressive approach could be reserved for symptomatic tumors, enlarging or functionally active lesions, or cases carrying *SDHB* or other higher-risk variants, in line with evolving European practice.

The main strength of this study is its large cohort of Chinese patients. Limitations include the absence of presurgical biochemical assessment and systematic evaluation of functional status due to the retrospective design, shorter follow-up times for the Chinese patients, and a selection bias due to referral practices, resulting in the relatively small number of jugulotympanic HNPGLs, as well as the absence of vagal HNPGLs, compromising the power of comparison between Chinese and European patients. Future studies are needed to investigate those subgroups of HNPGLs more closely. The observed differences between the two cohorts could also be biased by differences in clinical presentation, health-care–seeking behavior of patients, clinical practice, germline vs somatic genetic testing, in addition to ancestry. Additional limitations arise from differences in patient management between countries. In the Netherlands and Germany, MRI is the preferred choice of imaging modality to detect multifocality and additional tumors, whereas CT was predominantly used in the Chinese cohort. Whether this difference in imaging work-up contributed to the lower proportion of multilocal HNPGLs in the Chinese patients remains to be determined. Furthermore, clinical differences in selecting patients for surgery may contribute to higher recurrence rates in Europeans. In the Chinese cohort all patients underwent surgery, while this applied to only 54% of Europeans, who were followed by watchful waiting in 29% of cases. This may select for a more aggressive phenotype in those who receive surgery in Europe.

In summary, we have documented the clinical and genetic characteristics of HNPGLs in a large representative Chinese cohort. The genetic background of HNPGLs is characterized by a higher percentage of PVs in *SDHB* compared to Dutch/German Europeans and somewhat different clinical features of HNPGLs, most strikingly a younger age of diagnosis. It might be necessary to take ancestry into consideration in future clinical guidelines.

## Data Availability

The datasets generated during and/or analyzed during the current study are not publicly available but are available from the corresponding authors on reasonable request.

## Abbreviations

CT: computed tomography
HNPGL: head and neck paraganglioma
MRI: magnetic resonance imaging
NGS: next-generation sequencing
PGLs: paragangliomas
PNMT: phenylethanolamine *N*-methyltransferase
PV: pathogenic variant
SDH: succinate dehydrogenase
TH: tyrosine hydroxylase
VHL: von Hippel-Lindau
